# Association Between Celiac Disease and Cancer

**DOI:** 10.3390/ijms21114155

**Published:** 2020-06-10

**Authors:** Irene Marafini, Giovanni Monteleone, Carmine Stolfi

**Affiliations:** 1 Department of Systems Medicine, University of Rome “Tor Vergata”, 00133 Rome, Italy; irene.marafini@gmail.com (I.M.); gi.monteleone@med.uniroma2.it (G.M.); 2 Gastroenterology Unit, Policlinico Tor Vergata, 00133 Rome, Italy; 3 Division of Clinical Biochemistry and Clinical Molecular Biology, University of Rome “Tor Vergata”, 00133 Rome, Italy

**Keywords:** small bowel adenocarcinoma, T-cell lymphoma, colorectal cancer, gluten, refractory celiac disease, HLA-DQ2, HLA-DQ8, gluten-free diet

## Abstract

Celiac disease (CD) is a chronic enteropathy that develops in genetically susceptible individuals after the ingestion of gluten. There has been a substantial increase in CD prevalence in the last 50 years, and it is now estimated that this disease affects approximately 1% of the population in the Western world. In the large majority of cases, CD is a benign disease, characterized by the complete resolution of symptoms and a normal life expectancy after the onset of a gluten-free diet (GFD). However, failure to adhere to a strict GFD bears the risk of adverse events and increases mortality. A considerable number of studies have considered the possible association between CD and neoplasms. In particular, an increased risk of malignancies, such as cancers of the gastrointestinal tract and intestinal lymphomas, has been reported. In this review, we summarize and discuss the current evidence on the possible association between CD and cancer.

## 1. Introduction

Celiac disease (CD) is a chronic enteropathy that develops in genetically susceptible people after the ingestion of dietary gluten present in wheat, barley, and rye [1]. Despite CD is considered a public health problem worldwide, the exact global prevalence of CD is unknown as many patients remain undiagnosed for several years before receiving a correct diagnosis and appropriate treatment [2]. In a recent meta-analysis, CD prevalence worldwide based on serologic tests was 1.4%, whereas it was 0.7% based on biopsy results. The prevalence was higher in females compared to males and in children compared to adults [3].

CD develops from the encounter of an environmental factor (gluten) with a genetically predisposed individual (bearing human leukocyte antigen (HLA)-DQ2/HLA-DQ8 haplotypes), with the possible participation of other environmental co-factors [1]. For example, viral infections (such as those provoked by rotaviruses) would seem to increase the risk to develop CD in specific cohorts [4]. Changes in infant feeding practices have long been considered a triggering factor, but two prospective longitudinal studies in large cohorts of children denied this hypothesis [5,6]. In a prospective observational birth cohort study—called The Environmental Determinants of Diabetes in the Young (TEDDY)—evaluating the amount of gluten intake associated with CD autoimmunity, 6605 children, enrolled between 2004 and 2010, were followed up until September 2017 [7]. In this population, higher gluten intake within the first 5 years of age was associated with increased risk to develop CD in children bearing the predisposing HLA genotype (absolute risk difference, 7.2%) [7]. However, factors enabling the immune homeostasis to tip in favor of overt CD are still unknown as the majority of individuals with a predisposing genotype do not develop CD.

Three main features characterize the histopathology of CD: the increase of intraepithelial lymphocytes, crypt hyperplasia, and villous atrophy. Upon entering the lamina propria, gluten peptides are deamidated by the enzyme tissue transglutaminase 2 and then presented by antigen-presenting cells (HLA-DQ2/HLA-DQ8 positive) to CD4+ T (T-helper) cells [1]. T-helper cells drive a type 1 immune response and favor the activation of cytotoxic effector cells. This immune response is a powerful promoter of the expansion of cytotoxic intraepithelial lymphocytes and of the expression of natural killer (NK) receptors that enhance enterocyte apoptosis [8]. The ultimate mucosal damage is the result of a complex interaction between adaptive immunity and the effect of cytotoxic intraepithelial cells [9]. A recent study by Abadie and co-workers has further clarified such mechanisms by proposing the first pathophysiological mouse model of CD, in which the ingestion of gluten in an immunocompetent host promotes villous atrophy in a gluten- and HLA-DQ8-dependent manner [9]. The authors demonstrated that CD results from the complex interaction between several adaptive and innate immune pathways—all of them necessary to culminate in tissue destruction—and confirmed the crucial role of interleukin (IL)-15 in CD pathogenesis [9].

Clinical presentation of CD is largely variable as patients can be either asymptomatic or severely symptomatic [2]. In adults, diagnosis is based on measurement of serological anti-endomysial and anti-transglutaminase 2 antibodies, followed by histological confirmation with duodenal biopsy. HLA-DQ2/HLA-DQ8 genotyping is useful to rule out CD in high-risk individuals, but it is not required to make the diagnosis [10].

The only proven, worldwide-accepted treatment for CD is a strict, lifelong, gluten-free diet (GFD). All gluten-containing products should be avoided as even small amounts of gluten can be harmful. GFD compliance is often difficult for patients, in particular for teenagers and asymptomatic patients diagnosed with screening programs. Dietary compliance is routinely assessed during monitoring visits using standardized questions, and patients are followed-up through the measurement of serological antibodies at least once-a-year.

The vast majority of CD patients fully respond to GFD and have a normal life expectancy, without complications. However, older age, diagnostic delay, and poor adherence to GFD are risk factors to develop disease complications such as refractory celiac disease (RCD), enteropathy-associated T-cell lymphoma (EATL), and small bowel carcinoma (SBC) [11,12,13]. Accordingly, a number of studies have suggested an increased risk for certain types of cancer in CD patients. In this review, we report and discuss the available evidence on the association between CD and the risk of developing neoplasms. 

## 2. Refractory Celiac Disease

RCD is a rare complication of CD, characterized by persistence of malabsorption and villous atrophy despite strict adherence to GFD for at least 12 months [14]. Considering the rarity of this condition, before making a diagnosis of RCD, all other causes of malabsorption should be ruled out [14]. RCD is divided into two categories, namely RCD type 1 and RCD type 2, which differ in every aspect (clinical, histological, and molecular) (Table 1).

Thus, it is of paramount importance to distinguish between the two types of RCD as both prognosis and treatment differ as well [14].

RCD must be considered when addressing the issue of cancer risk in CD patients, as RCD type 2 (characterized by aberrant proliferation of monoclonal lymphocytes) is considered as a pre-lymphoma and even a low-grade lymphoma, due to the high risk to evolve in overt intestinal lymphoma [14]. In the majority of cases, RCD is diagnosed after 50 years of age. Pathogenesis of RCD type 1 is mostly unknown and sometimes it is difficult to differentiate this condition from slow responders that eventually normalize intestinal mucosa, an event that may take more than one year. On the other hand, some recent papers have elucidated the mechanisms underlying RCD type 2 [14,15,16]. As mentioned above, RCD type 2 is characterized by aberrant T cells that do not express surface CD3 or CD8 but, instead, express intracellular CD3 with a monoclonal T cell receptor (TCR) rearrangement [14]. These cells express NK surface markers and respond well to IL-15 [16,17], which is considered one of the main drivers of tissue damage in RCD. Moreover, they arise from a small cluster of cells present in the gut of healthy individuals and show augmented responsiveness to IL-15 due to gain-of-function mutations occurring in the genes for Janus kinase (JAK) and Signal transducer and activator of transcription (STAT)3 [17]. IL-15 production, which is increased in the gut of RCD patients [18], favors the production of interferon (IFN)-γ as well as the expansion of the aforementioned abnormal cytotoxic T cells. The same mutations underlying RCD type 2 seem to be at the origin of EATL [19]. Additionally, in RCD, inflammation is sustained by defects in counter-regulatory mechanisms, such as the transforming growth factor (TGF)-β pathway [20].

Both types of RCD present with signs of malabsorption (diarrhea and weight loss) and consequent malnutrition (hypoalbuminemia, vitamin, and iron deficiency) [21]. In the face of suspected RCD, the first step consists of confirming the underlying diagnosis of CD [10]. Once other causes of villous atrophy are excluded, upper endoscopy with duodenal biopsy should be performed to confirm the diagnosis and discriminate between RCD type 1 and type 2. Histologically, RCD type 1 is indistinguishable from untreated CD, whereas RCD type 2 is diagnosed for the presence of more than 20% aberrant, clonal T lymphocytes [22,23]. Patients with RCD type 2 should be monitored for the development of EATL with repeated endoscopies and the use of computed tomography (CT) or magnetic resonance (MR) enterography and positron emission tomography (PET) when appropriate.

In RCD type 2 patients, a GFD is recommended but the effectiveness is unclear. Other treatments are azathioprine, cyclosporine, corticosteroids (including budesonide), infliximab, mesalamine, tioguanine, cladribine, and autologous hematopoietic stem cell transplantation. Considering the role of IL-15 in the pathogenesis of RCD, an antibody targeting this cytokine has recently been developed [24]. In a phase 2a, placebo-control clinical trial, AMG 714, an anti-IL-15 monoclonal antibody, failed to reach the primary end-point, which was the reduction of aberrant intraepithelial lymphocyte from baseline [25]. However, the reported positive effects on symptoms and other secondary end-points would suggest further exploration of anti-IL-15-based therapies in RCD [25].

## 3. Celiac Disease and Risk of Malignancies

In the last decades, a large number of studies prompted to investigate the risk of developing malignancies in CD patients. Taking into account the most relevant studies, the overall cancer risk would seem higher in most of the cases as compared to the general population (Table 2). However, it is worth noting that most of these surveys were limited by the small number of cancers analyzed and by the fact that were conducted in referral centers. Thus, such biases may have led to an overestimation of cancer risk in CD.

In a Swedish study by Askling and co-workers, 11,019 patients with a diagnosis of CD were analyzed [11]. Among these patients, 8012 (73%) were under 19 years old. In this cohort, the overall risk of developing a malignancy was very low. However, the fact that such patients were not followed long enough to witness the development of cancers may likely explain the documented low risk. Moreover, the risk of malignancies decreased with follow-up and, after 10 years, CD patients were not anymore at increased risk compared to the general population. In this study, the first year of follow-up after CD diagnosis, when the most diagnoses of cancers are made, was excluded [11]. 

Two British studies found, respectively, a very low risk and an absence of risk of malignancies in CD [26,37]. Elfstrom and colleagues found no increased risk of overall cancer in CD. Cancer risk in CD patients was concentrated within the first year of diagnosis [12]. In another Swedish cohort, CD patients were at 55% increased risk for dying of any malignancy [11]. All these analyses were made in patients with diagnosed CD. There are few studies on the risk of malignancies on undiagnosed CD, a sub-population in which it is very difficult to calculate a precise risk [38,39,40]. 

As far as lymphoproliferative malignancies are concerned, many studies have found a significantly increased risk in CD patients. The study by Elfstrom and colleagues found a risk of 2.82 of lymphoproliferative malignancies that decreased during follow-up [27]. Other studies reported a 2- to 5-fold increase risk of Hodgkin lymphoma (HL) in CD patients compared to the general population [11,12,27,28]. Patients with CD, similarly to other autoimmune/inflammatory conditions, have also an increased risk of non-Hodgkin lymphoma (NHL). The most frequently affected site is the intestine [27,41,42]. The highest relative risks are for T cell NHL, but also B cell NHL are described in CD. This is in accordance with the relevance of B cells in CD-pathogenesis, as antigen-presenting cells and immunoglobulin producers [43]. The most likely explanation for this association between lymphoma and CD is chronic inflammation. This hypothesis is also supported by the evidence that patients with villous atrophy have statistically a higher risk of lymphoproliferative disorders as compared to patients with crypt hyperplasia and the increase of intraepithelial lymphocytes. In this context, the role of a GFD would seem to be of benefit. 

CD is associated with a modest long-term increased risk of mortality. In a population-based cohort study conducted in Sweden including 49,829 patients diagnosed between 1969 and 2017, a diagnosis of CD was associated with a small but statistically significant increased mortality risk as compared with the general population. However, such risk was predominantly attributable to cardiovascular and respiratory diseases and not to cancers [44]. Recently, Koskinen and colleagues analyzed a cohort of 12,803 CD patients and documented that the overall mortality from all malignancies or cardiovascular diseases was not increased. On the other hand, the mortality from lymphoproliferative diseases was increased (HR 2.36), although lower than previously reported [45].

## 4. Association Between CD and Individual Cancers

### 4.1. Enteropathy-Associated Lymphoma 

The first association between CD and lymphoma goes back to 1937 when Fairley and Mackie described six patients with intestinal lymphoma and steatorrhea [46]. Since then, other reports followed and in 1986 the term enteropathy-associated T cell Lymphoma (EATL) was firstly used to identify the rare form of high-grade T-cell NHL of the upper small intestine, specifically associated with CD [47]. EATL is a rare form of cancer that predominantly occurs in patients in the seventh decade of age. Usually, EATL arises in patients with a diagnosis of CD, either pre-existing or made concomitantly [48,49]. EATL has an incidence rate of approximately 0.10 cases per 100,000 inhabitants/year, with a large prevalence in males compared to females [50]. The tumor is more frequently localized in the jejunum compared to the ileum and it is often multifocal with ulcerative lesions. EATL immune phenotype is characterized by the clonal proliferation of intraepithelial lymphocytes TCR α/β positive [51,52]. Several histological varieties are described, but most cases consist of medium-to-large-sized cells with a pleomorphic appearance and an increased mitotic index [53]. In many cases, but not necessarily, EATL is the end stage of RCD type 2. Since 2008, the World Health Organization (WHO) has identified a minority (<20%) of EATL that meets specific molecular criteria (for example, the expression of CD56) and is less often associated with CD [54].

A large study on CD-associated malignancy demonstrated that CD-associated lymphoma risk may not be as high as previously noted [11] (Table 3).

However, despite the magnitude of risk remain debatable, the unequivocal association between CD and lymphoma remains. This same study gives information on the positive role of GFD in the prevention of EATL development. Many studies provided evidence that prompt diagnosis and strict adherence to GFD may decrease cancer risk and mortality [11,55,56,57,58]. However, both EATL and other malignancies have been described in patients on a GFD as well [13]. This latter finding, which apparently denies the beneficial effect of a GFD, could be explained by the fact that these patients have been exposed for too many years to a gluten-containing diet in face of few years of GFD, and this could be insufficient to revert the effect of longstanding gluten exposure. 

Some patients have disseminated disease at diagnosis, with extra-intestinal localizations. Symptoms include abdominal pain, diarrhea, weight loss, fever, lymphoadenopaty, hepatomegaly, and palpable abdominal mass. Upper and lower endoscopy, enteroscopy, and CT and MR enterography are part of the diagnostic work-up to diagnose and stage EATL. In some patients, laparoscopy can be necessary to reach a final diagnosis. The therapeutic management of EATL is particularly difficult and survival is poor (13% at 30 months) [48,59].

Some authors suggest that CD patients are at increased risk of malignant lymphomas other than EATL. In a large prospective cohort of CD patients, a six-fold increase in overall lymphoma risk was observed [11]. Histopathological analysis of 58 patients included in this study demonstrated that non-intestinal B cell and T cell NHLs constituted the majority of CD-associated malignant lymphomas [29]. Larger studies are, however, needed to confirm these observations.

### 4.2. CD-Associated Small Bowel Carcinoma

SBC is an extremely rare neoplasm that accounts for less than 5% of all gastrointestinal cancers [60]. It may occur either as a sporadic tumor or associated with predisposing inflammatory conditions. In Europe, SBC has an estimated incidence rate of 3600 new cases/year, with a median age in the seventh decade [60].

Many epidemiological studies and meta-analysis suggest that CD patients have a higher risk to develop SBC compared to the general population [13,30] (Table 4).

Accordingly, among all SBC, 13% are associated with a diagnosis of CD [48]. The first case of SBC in CD was described in 1972 and, from that on, many other reports followed up [61].

CD patients with a diagnosis of SBC usually have a median age between 53 to 62 years old. Risk factors for CD-associated SBC are not completely clear, but strict adherence to the GFD seems to have a protective role. In a large cohort study conducted in Sweden, the increased risk to develop gastrointestinal cancers in CD patients was completely abolished after one year from the diagnosis, suggesting a beneficial role of controlling intestinal inflammation [12]. On the other hand, SBCs have also been described in CD patients on a strict GFD, thus highlighting the importance of other predisposing factors in the development of this complication. It is noteworthy that the median age at diagnosis for CD patients with SBC was significantly higher than the median age at diagnosis for CD patients without malignancies [62,63]. This observation suggests that the diagnostic delay could play a role not only in the development of refractoriness and intestinal lymphoma but also in CD-associated SBC. Overall, apart from the age at diagnosis, a delay in the identification of CD, and poor adherence to the GFD, there are no other identified risk factors for the development of CD-associated SBC.

Considering the rarity of this tumor, molecular data are scarce. However, Vanoli and co-workers identified specific features of CD-related SBC in a large case series [62]. Compared to sporadic SBC and Crohn’s disease-associated SBC, CD-SBC was characterized by frequent microsatellite instability (MSI) and high density of tumor-infiltrating T lymphocytes [62]. The same group recently reported that two main molecular subtypes characterize CD-related SBC, the MSI-immune subtype, and the mesenchymal subtype, with the latter associated with prominent TGF-β production and matrix remodeling [64].

Some reports suggest that CD-associated SBC arise from the classic “adenoma-to-carcinoma sequence”, although this hypothesis is still highly debated [65]. In CD patients, the most frequently affected site is the jejunum as compared to the duodenum and ileum [66]. Time of onset and clinical presentation are largely variable. Some cohort studies reported a median onset time ranging from 1.4 to 17 years from CD diagnosis, whereas in some cases SBC and CD can be diagnosed at the same time [62]. Patients can present with direct or indirect signs of intestinal bleeding, such as overt hemorrhage, melena, coffee ground vomiting, and anemia, with obstructive symptoms (e.g., nausea, vomiting, abdominal pain), or with intussusception or perforation [67]. In patients with known CD, in the presence of the abovementioned symptoms, SBC (together with other CD-related complications) must be suspected and investigated. Esophagogastroduodenoscopy with biopsy is usually the first exam to perform to identify lesions proximal to the Treitz ligament. However, since most CD-associated SBC are localized in the jejunum, other techniques, such as enteroscopy, CT enterography, and MR enterography, are often required [68]. On the other hand, small bowel capsule endoscopy is not recommended due to the risk of capsule retention and the impossibility to collect mucosal samples.

SBC prognosis is generally extremely poor. A retrospective study analyzing the records of 491 patients with a diagnosis of SBC, both sporadic and associated with predisposing conditions such as CD, showed a median overall survival of 20.1 months, with a 5-year overall survival of 26% [69]. Age at diagnosis, stage of the disease, and the presence of lymph nodes or distant metastases were the factors that most correlated with a poor outcome [70,71]. When CD-associated SBC was specifically assessed, survival was better compared both to sporadic SBC and to Crohn’s disease-associated SBC: two separate cohorts demonstrated a 5-year overall survival of 64.2% and 83% for CD-associated SBC [62,72]. Patients with diffuse-, mixed-, and solid-type histology tended to have a worse prognosis compared to glandular-type and medullary-type cancers [73,74]. Molecular subtypes have also been associated with prognosis: SBC with microsatellite instability are more likely indolent, whereas mesenchymal subtypes present worse tumor behavior [64]. Considering the rarity of CD-associated SBC, all therapeutic recommendations derive from the treatment of sporadic SBC. Surgery is the mainstay and can be curative only in the early stages of the disease, while surgery plus adjuvant chemotherapy is reserved for advanced stages. There is a large variety of medical therapies (e.g., classic chemotherapy, novel immune- and molecular-targeted therapies) used to treat solid tumors including SBC for which the reader is directed toward excellent reviews [75,76]. Among the possible therapeutic target in SBC, there is the programmed cell death protein-1 (PD-1)/programmed cell death ligand 1 (PD-L1) pathway. This pathway was recently evaluated in a large series of SBC. PD-L1 was highly expressed in CD SBC compared to sporadic SBC, and PD-1-positive immune cells were largely present in CD SBC compared to the sporadic ones [77]. These findings further support the possibility of the use of checkpoint inhibitors in CD-associated SBC. 

### 4.3. Other CD-Associated Malignancies

Although the aforementioned studies clearly indicate that the risk to develop EATL and SBC is higher in CD patients as compared to the general population, whether CD patients are more susceptible to develop other malignancies is still under debate. In this context, some authors reported an increased risk for CD patients to develop pharyngeal and esophageal carcinomas [78,79] (Table 5).

Using a Swedish registry of around 12,000 CD patients, Askling and colleagues reported an increased risk to develop malignant lymphoma, SBC, oropharyngeal, esophageal, large intestinal, hepatobiliary, and pancreatic carcinoma [11]. In the same paper, the authors reported that CD patients had a decreased risk of breast cancer, a finding confirmed by other population studies [26]. The protective role of CD toward breast cancer has been reported by many different studies, although the reasons for this negative association are not clear. A large study by Ludvigsson confirmed the protective effect of CD, but with borderline significance [31]. 

As already mentioned above, in a more recent Swedish cohort study of CD patients, the risk of any gastrointestinal (GI) cancer decreased over time: during the first year after diagnosis and initial biopsy, CD was associated with a 5.95-fold increase in risk of incident GI cancer of any type, whereas one year after the diagnosis, patients were not at increased risk [30]. Overall, after the first year of diagnosis, CD patients seemed to have a lower absolute risk to develop cancer [12]. The highest relative risk for GI cancer in CD was seen for SBC and pancreatic adenocarcinoma [12]. There was an 8-fold increase in colorectal cancer (CRC) in the first year of diagnosis that was abolished after one year [12]. Misdiagnosis of CD in patients that eventually resulted to have cancer is a possible explanation for the increased risk observed within the first year of diagnosis. Indeed, the overall risk of CRC in CD is comparable to the general population [12]. A case-control study conducted in 2010 found no association between colonic adenomas and CD [32]. Another multicenter, retrospective case-control study, within four community hospitals, demonstrated that CD was not associated with an increased risk of CRC [33]. Finally, a population study conducted in Italy reported that CD patients have even a lower risk to develop CRC as compared to the general population [80].

The literature regarding the risk of melanoma in CD is conflicting. Two studies found no association [11,26], while an increased risk of melanoma was described in a cohort of US patients affected by CD [13]. In a population-based study conducted in 2014 in Sweden, in which 29,028 CD patients were each matched with 5 controls, no association between these two diseases was reported [34]. Considering these results, it is likely there is no causal relationship between melanoma and CD. 

The studies on the risk of developing thyroid cancer in CD are conflicting as well. In 2006, Kent and co-workers identified an increased risk of papillary carcinoma of the thyroid (standard morbidity ratio of 22.52) in a US cohort of CD patients [81]. According to these data, an Italian study population including 1757 patients diagnosed with CD between 1982 and 2006 demonstrated a 2.5-fold increased risk of papillary cancer of thyroid [35]. However, only 6 thyroid cancers were identified during the study. On the other hand, Ludvigsson identified 15 thyroid cancers out of 29,074 patients with CD (HR 0.6) [36]. Collectively, these data are still inconclusive and no formal association between CD and thyroid cancer can be made. 

## 5. Conclusions

The available data suggest that adults with CD have an overall risk of developing intestinal lymphoma and SBC slightly increased as compared to the general population. Besides these two neoplasms, there is not sufficient evidence so far to suggest a higher prevalence of other malignancies in CD patients. Data on malignancies in children with CD are scarce. Two large studies did not find any increased risk of malignancy in children diagnosed with CD [11,82]. Only the study by Solaymani-Dodaran and colleagues found an excess of mortality due to cancer in children with CD [83]. However, given the low number of events reported (5 deaths), data must be taken with caution.

Overall, the risk of developing EATL and SBC is very small in humans. Despite that, as these types of cancer bear a poor prognosis, strategies aimed at reducing their incidence should be followed. So far, only adherence of CD patients to a GFD would seem to reduce the risk of these rare, though very aggressive, forms of cancer. In support of this hypothesis is the fact that children with CD do not have an increased risk of cancers in later life, further underlining the beneficial effect of a GFD [11,84]. Nevertheless, the effect of a GFD in preventing/reducing the risk of developing malignancies in CD patients is still debated. As the non-adherence and/or non-responsiveness to a GFD may lead to chronic inflammation of the small bowel, it is tempting to speculate that a gluten-containing diet in CD patients may promote the activation of immune/inflammatory signals and ultimately favor the onset/progression of lymphomas and SBCs. On the other hand, as CD patients drastically modify their dietary habits following the diagnosis of the disease, such changes could someway influence the risk of developing malignancies.

In conclusion, diversely to the increase of the awareness about CD pathogenesis that occurred in the last decade, as well as the reduction of the diagnostic delay, we still have poor knowledge about the risk factors/biological links that may contribute to the development of CD-associated neoplasms. Further mechanistic studies in experimental models as well as multicenter observational cohort studies, conducted not only in Western and/or westernized countries, would help clarify these issues.

## Figures and Tables

**Table 1:** Differences among CD, RCD type 1, RCD type 2, and EATL.

Examinations	CD	RCD Type 1	RCD Type 2	EATL
*Disease type*	Chronic enteropathy triggered by dietary gluten	Persistence of gluten-independent villous atrophy	Low-grade lymphoma	High-grade lymphoma
*Lymphocytes*	Increased IEL CD3+ with polyclonal TCR	Increased IEL CD3+ with polyclonal TCR	Increased IEL CD3- with monoclonal TCR	Atypical IEL with monoclonal TCR
*Clinical course*	Indolent	Rather indolent	Aggressive	Highly aggressive

Abbreviations: CD: celiac disease; RCD: refractory CD; EATL: enteropathy associated T cell lymphoma; SBC: small bowel carcinoma; IEL: intraepithelial lymphocytes; TCR: T cell receptor.

**Table 2:** Most relevant studies examining cancer risk in CD patients.

First Author	Year	Cancer Type	Cohort	Number of Patients	Notes	Ref.
**Askling**	2002	Malignant lymphoma Small-intestinal lymphoma Oropharyngeal Esophageal Large intestine Hepatobiliary Pancreatic	Swedish	11,019	The overall cancer risk is only moderately increased	[11]
**Askling**	2002	Breast	Swedish	11,019	-	[11]
**Card**	2004	Breast	British	869	-	[26]
**Elfstrom**	2012	Esophageal Gastric Small bowel Colon Rectal Liver Pancreatic	Swedish	28,882	The increased risk is limited to the first year after CD diagnosis	[12]
**Elfstrom**	2011	NHL Hodgkin lymphoma	Swedish	28,989	The risk was lower in patients with latent CD	[27]
**Goldacre**	2008	NHL	British	1997	-	[28]
**Smedby**	2005	B cell NHL Non-intestinal lymphoma	Swedish	11,650	44% of patients with B cell NHL had a history of autoimmune diseases other than CD	[29]
Han *	2015	Small bowel Esophageal	Meta-analysis (17 studies)	-	-	[30]
**Ludvigsson**	2012	Breast Endometrial Ovarian	Swedish	17,852	-	[31]
**Lebwohl**	2010	Colonic adenoma	US	180	-	[32]
**Pereyra**	2013	Colorectal	Argentine	118	-	[33]
**Lebwohl**	2014	Melanoma	Swedish	29,028	-	[34]
**Volta**	2011	Papillary thyroid	Italian	1757	Only 6 cases of thyroid cancers were identified	[35]
**Ludvigsson**	2013	Thyroid	US	606	-	[36]

Pink background = increased risk; green background = reduced risk; grey background = no risk change; * indicates that the study is a meta-analysis. Abbreviations: NHL, non-Hodgkin lymphoma; CD, celiac disease, US, United States.

**Table 3:** Studies examining lymphoma risk in CD patients.

Cancer Type	Cohort	First Author	Year	Ref.
**NHL**	British	Holmes	1989	[55]
**Malignant lymphoma**	British	Logan	1989	[56]
**NHL**	Finnish	Collin	1994	[57]
**NHL**	Italian	Corrao	2001	[58]
**Malignant lymphoma** **Small-intestinal lymphoma**	Swedish	Askling	2002	[11]
**NHL**	US	Green	2003	[13]
**B cell NHL** **Non-intestinal lymphoma**	Swedish	Smedby	2005	[29]
**NHL**	British	Goldacre	2008	[28]
**NHL** **Hodgkin lymphoma**	Swedish	Elfstrom	2011	[27]

Pink background = increased risk; grey background = no risk change; Abbreviations: NHL, non-Hodgkin lymphoma; CD, celiac disease, US, United States.

**Table 4:** Studies examining small bowel carcinoma risk in CD patients.

Cancer Type	Cohort	First Author	Year	Ref.
**Small bowel**	British	Kenwright	1972	[61]
**Small bowel**	British	Howdle	2003	[48]
**Small bowel**	US	Green	2003	[13]
**Small bowel**	Swedish	Elfstrom	2012	[12]
**Small bowel**	Meta-analysis (17 studies)	Han *	2015	[30]

Pink background = increased risk; * indicates that the study is a meta-analysis. Abbreviations: CD, celiac disease; US, United States.

**Table 5:** Studies examining the risk of developing other malignancies in CD patients.

Cancer Type	Cohort	First Author	Year	Ref.
**Large intestine**	Swedish	Askling	2002	[11]
**Colonic adenoma**	US	Lebwohl	2010	[32]
**Colon** **Rectal**	Swedish	Elfstrom	2012	[12]
**Colorectal**	Argentine	Pereyra	2013	[33]
**Colon**	Italian	Volta	2014	[80]
**Esophageal**	Swedish	Askling	2002	[11]
**Esophageal**	US	Green	2003	[13]
**Esophageal**	Swedish	Elfstrom	2012	[12]
**Esophageal**	Meta-analysis (17 studies)	Han *	2015	[30]
**Breast**	Swedish	Askling	2002	[11]
**Breast**	British	Card	2004	[26]
**Breast**	Swedish	Ludvigsson	2012	[31]
**Papillary thyroid**	US	Kent	2006	[81]
**Papillary thyroid**	Italian	Volta	2011	[35]
**Thyroid**	US	Ludvigsson	2013	[36]
**Pancreatic**	Swedish	Askling	2002	[11]
**Pancreatic**	Swedish	Elfstrom	2012	[12]
**Melanoma**	US	Green	2003	[13]
**Melanoma**	Swedish	Lebwohl	2014	[34]
**Oropharyngeal** **Hepatobiliary**	Swedish	Askling	2002	[11]
**Gastric** **Liver**	Swedish	Elfstrom	2012	[12]
**Endometrial** **Ovarian**	Swedish	Ludvigsson	2012	[31]

Pink background = increased risk; green background = reduced risk; grey background = no risk change; * indicates that the study is a meta-analysis. Abbreviations: CD, celiac disease, US, United States.
